# Integrating transcriptome and metabolome analyses of the response to cold stress in pumpkin (*Cucurbita maxima*)

**DOI:** 10.1371/journal.pone.0249108

**Published:** 2021-05-06

**Authors:** Fengmei Li, Xiuping Lu, Pengfei Duan, Yanjiao Liang, Jian Cui

**Affiliations:** 1 Shandong Provincial Key Laboratory of Biochemical Engineering, College of Marine Science and Biological Engineering, Qingdao University of Science & Technology, Qingdao, Shandong, China; 2 Qingdao Institute of Agricultural Science Research, Qingdao, Shandong, China; Universidade de Lisboa Instituto Superior de Agronomia, PORTUGAL

## Abstract

*Cucurbita maxima* belong to the genus *Cucurbita* and are of nutritional and economic importance. Physiological activity, transcriptome, and metabolome analyses of leaf samples from the *C*. *maxima* inbreding line IL7 treated at 5 °C and 25 °C were performed. Cold stress resulted in a significant increase in the malondialdehyde content, relative electrical conductivity, soluble protein, sugar content, and catalase activity. A total of 5,553 differentially expressed genes were identified, of which 2,871 were up-regulated and 2,682 down-regulated. In addition, the transcription of differentially expressed genes in the plant hormone signal transduction pathway and transcription factor families of AP2/ERF, bHLH, WRKY, MYB, and HSF was activated. Moreover, 114 differentially expressed metabolites were identified by gas chromatography time-of-flight mass spectrometry, particularly through the analysis of carboxylic acids and derivatives, and organooxygen compounds. The demonstration of a series of potential metabolites and corresponding genes highlighted a comprehensive regulatory mechanism. These findings will provide novel insights into the molecular mechanisms associated with the response to cold stress in *C*. *maxima*.

## Introduction

Low temperature is one of the major environmental factors limiting agricultural productivity and the geographic distribution of many plant species [1]. During early spring, low temperatures are not conducive to plant survival and development, leading to a decline in fruit yield and quality. Some plant species have developed a remarkable ability to adapt to low temperatures [2]. Through intensive research, a variety of mechanisms employed to respond to temperature have been identified, but the way plants sense low temperature remains unknown [3].

After plant exposure to cold stress, a series of physiological and biochemical changes at the molecular and cellular levels are induced to adapt and survive under cold environments [4,5]. These changes include increased accumulation of antioxidant enzymes and non-enzymatic molecules which may neutralize or counteract the harmful effects of reactive oxygen specie (ROS) and protect the plants from cold stress [6]. In the process of cold stress response, a series of osmoregulatory metabolites including soluble sugar (e.g., sucrose, glucose and galactose) and low molecular weight compounds (such as proline, glycine betaine), these substances can help alleviate osmotic stress, expansion, water uptake and metabolic activity of plant cells [7,8]. To date, considerable research has been conducted on the physiological and biochemical mechanisms of plant cold resistance. For example, physiological analyses of *Santalum album* L. leaves treated at 4 °C for 48 h demonstrated that cold stress induces the accumulation of malondialdehyde (MDA), proline, and soluble sugar (SS), while increasing the levels of superoxide dismutase (SOD) and peroxidase (POD) activity [9]. Ma et al. analyzed the physiological response of *Brassica rapa* L. held at 4 °C for 24 h to reveal that the POD and catalase (CAT) activity, SS content, and MDA content of the cultivars Longyou-7 and Lenox increased under cold stress [10]. These results indicated that different plants exhibit different physiological responses to cold stress.

Plant transcriptomics and metabolomics are now used to explore the genes and metabolites that enable cold survival. These resources are greatly expanding our understanding of important molecular processes. The study of global gene expression using RNA-Seq can help researchers to unveil the regulatory genes and key pathways that may induce cold resistance. Hence, an increasing number of cold sensitive genes and transcription factors have been identified and analyzed in plants under cold stress [9,11–14]. WRKY, NAC, MYB, AP2/ERF, and bZIP were the most abundant transcription factor families under cold stress. These transcription factor families are known for role in ABA-induced signaling pathways under low temperature [15,16].

Moreover, multiple pathways have been recognized to contribute directly to the cold stress response in plants. Most pathways such as flavonoid biosynthesis, phenylpropanoid biosynthesis, plant hormone signal transduction, cutin, suberine and wax biosynthesis, pentose and glucuronate interconversions, phenylalanine metabolism and starch and sucrose metabolism were significantly enriched KEGG in rubber tree (*Hevea brasiliensi*s) after cold stress [17]. The plant hormone signal transduction pathway has been found to provide tolerance to cold stress in several species including *Arabidopsis thaliana* [12] and Strawberry (*Fragaria × ananassa*) [13,14]. Recently, the best approach has been to integrate transcriptomics and metabolomics methods to reveal the complex reaction process that occurs under low temperature stress and provide more information about this response. In recent years, this integrative method has become a useful tool for producing a large amount of molecular information about global cellular events and has been used to study peach, tobacco, seaweed, tomato, and grape, etc [11,13,16–18]. Although several molecular mechanisms related to cold stress have been revealed in these plants, little is known about the response of pumpkin, *Cucurbita maxita*, to cold stress.

In agricultural production, *C*.*maxita* is an important horticultural crop [19] and it is worldwide-cultureted crop because of its remarkable nutritious and economic value. Moreover, *C*. *maxita* is often used as important rootstocks for grafting watermelon, cucumber, and melon, it can increase tolerance to cold stress [20]. Although seven cold stress-related glutathione transferase (GST) genes were explored in the cold-tolerant line (*C*. *maxima*) and are putative candidates for use in breeding cold-tolerant crops varieties [21], the molecular studies of pumpkin are still in their infancy. Studies of differences in transcriptional and metabolic levels of pumpkin under cold stress can improve our understanding of cold stress responses mechanism.

## Materials and methods

### Plant material, cold treatments, and physiological analyses

The *C*. *maxima* inbred line IL7, provided by the Qingdao Academy of Agricultural Sciences of Shandong Province, China, was used in this study. IL7 has been purified by more than eight consecutive generations of self-pollination.

Seeds were sown in a universal horticultural peat-based soil mixture. Plants were grown for 2 weeks in cultivation chambers at 25/19 °C (day/night) and 80% relative humidity; the digital illumination reached 24,000 Lux. These plants were grown inside cultivation chambers (Ningbo Safe Instrument Co., Ltd., Ningbo, China) set at 25 °C (control). After 3 weeks of growth, corresponding to the 3–4 leaf stage, cold treatment at 5 °C (16 h of light/8 h of dark) was applied and visible disease symptoms were confirmed after 24 h. Leaves were collected 24 h after the onset of the cold stress treatment and prepared in triplicate.

### Physiological analyses

The experiments were conducted in a completely randomized block design with three replications so that each data point was the average of three independent samples. Membrane permeability, expressed by relative electric conductivity (REC), was determined by a modified method [22]. The Soil Plant Analysis Development (SPAD) value, the index of total chlorophyll, was detected using a portable chlorophyll meter (SPAD-502 Plus, Minolta Camera Co. Ltd., Japan) [23]. The determination of the soluble sugar (SS) and soluble protein contents, SOD activity, and MDA content was carried out using a kit according to the instructions provided by the Nanjing Jiancheng Biology Engineering Institute. The POD and CAT activities were detected using a kit according to the instructions provided by the Suzhou Comin Biotechnology Co., Ltd.

### Total RNA extraction, library preparation, and Illumina sequencing for transcriptome analysis

Total RNA was extracted from young *C*. *maxima* leaves of the control (untreated) and cold-stressed (treated) (5 °C, 24 h) plants using the mirVana miRNA Isolation Kit (Ambion). Three biological replicates were used and the RNA was stored at -70 °C until the RNA-seq library preparation was complete. Real biological variability can be better simulated and statistical accuracy improved by using biological replicates. The integrity of the total RNA from the *C*. *maxima* leaves was analyzed using an Agilent 2100 Bioanalyzer system (Agilent Technologies, Santa Clara, CA, USA). We pooled the high-quality reads from the samples to perform *de novo* transcriptome assembly. Samples with an RNA integrity number ≥ 7 were subjected to subsequent analyses. A TruSeq Stranded mRNA LTSample Prep Kit (Illumina, San Diego, CA, USA) was used to construct the libraries. The libraries were sequenced using an Illumina HiSeqTM 2500 sequencing platform, and 150 bp paired-end reads were generated.

### Identification and annotation of DEGs for transcriptome analysis

Raw reads were removed with poly-N and low-quality reads were removed to obtain clean reads. The RNA-Seq samples were then mapped to the *C*. *maxima* genome (https://www.ncbi.nlm.nih.gov/assembly/GCF_002738345.1/) as the reference genome using Tophat [24]. HTSeq software (Heidelberg, Germany) was used to obtain the read counts of each gene [25] and Cufflinks [26] software computed the gene expression fragments per kilobase of transcript per million mapped reads (FPKM) values, and the read counts for each gene were obtained from the HTSeq-count [25]. The linear association between the gene expression levels of the sequenced samples was evaluated using Pearson’s correlation coefficient. The distances between the samples were calculated using clustering methods to perform principal component analyses of the gene expression levels.

For quantification at the transcript level, the FPKM and read count value of each transcript were calculated using bowtie2 [27] and eXpress. The DEGs were identified using the estimate Size Factors and negative binomial (nbinom) tests in the DESeq function of R package [28]. The software enabled us to obtain the number of reads of a gene in each sample, normalize the data using DESeq, and use the nbinom test function to calculate the *p*-value and fold change values. RNA-seq means from the same gene in two samples were considered to indicate statistically differential expression when the *p*-value was <0.05 and fold change was >2. A hierarchical cluster analysis of DEGs was performed to explore the transcript expression pattern. Both a gene ontology (GO) analysis and Kyoto Encyclopedia of Genes and Genomes (KEGG) pathway enrichment analysis of the DEGs were respectively performed using R, based on the hypergeometric distribution. The results were reassembled using the Tophat-Cufflinks platform [26]. The results from the Cuff compare software were compared with the gene annotation information from the reference to obtain sequenced gene structure extensions and identify new transcripts. The alternative splicing analysis of differentially regulated transcripts, isoforms, or exons was performed using Asprofile [28].

### Verification of differential expression using real-time quantitative reverse transcription-PCR

The total RNA was extracted from the leaves of pumpkin seedlings treated with cold stress for 0 or 24 h as described above. Quantification was performed using a two-step reaction process involving reverse transcription (RT) and PCR. In the first step, 0.5 μg RNA and 2 μL of 4 × gDNA wiper Mix were added to nuclease-free H_2_O to give a final volume of 8 μL. The reaction was carried out on a GeneAmp^®^ PCR System 9700 (Applied Biosystems, USA) for 2 min at 42 °C. Then, 2 μl of 5 × HiScript II Q RT SuperMix II were added. The reaction was then carried out for a further 15 min at 50 °C, followed by 5 s at 85 °C. The reaction mixture was then diluted in nuclease-free H_2_O and stored at -20 °C. Real-time PCR was performed using a LightCycler^®^ 480II real-time PCR instrument (Roche, Switzerland). The reactions were conducted in a mixture volume of 10 μl that included 5 μl of 2 × ChamQ SYBR qPCR Master Mix. There were three biological replicates of each sample. The PCR thermocycling program consisted of an initial incubation at 95 °C for 30 s, followed by 40 cycles at 95 °C for 10 s, and 60 °C for 30 s. The β-actin gene was used as an internal control, and gene specific primers were designed using Primer Premier 5.0 software. The primers are presented in S1 Table. After normalizing the mRNA expression level, it was calculated using the 2^-ΔΔCt^ method [29].

### Metabolites

Ten samples from young leaves of the control (untreated) and cold treatment (5 °C, 24 h) groups were analyzed using a gas chromatography time-of-flight mass spectrometry system. The freeze-dried samples were crushed at 45 Hz for 4 min using a mixer mill. A 50 mg aliquot of sample was added to a 1.5 mL Eppendorf tube, and the sample was extracted by adding 1mL of 75% methanol and placing the tube on a shaker overnight at 4 °C. Then, the samples were centrifuged for 15 min (12,500 rpm, 4 °C). The resulting supernatants were transferred to 2 mL sample vials and stored at -80 °C until the ultra-high performance liquid chromatography (UHPLC)-tandem mass spectrometry (MS/MS) analysis. A quality control sample was prepared by mixing equal aliquots of the supernatants from all samples.

### LC-MS analysis

The UHPLC separation was performed using a Waters ACQUITY UPLC HSS T3 column (100 × 2.1 mm, 1.8 μm). We used 0.1% formic acid in H_2_O as mobile phase A and acetonitrile as mobile phase B. During the LC/MS experiment, the MS/MS spectra were acquired by a Triple TOF 6600 mass spectrometer in the information-dependent acquisition (IDA) mode.

In the IDA mode, the data acquisition software (Analyst TF 1.7, AB Sciex) collects and triggers the acquisition of MS/MS spectra based on MS data, depending on preselected criteria. For each cycle, 12 precursor ions with intensities greater than 100 were selected. The collision energy (CE) was 30 V, and the product ion accumulation time of each 12 MS/MS events was 50 ms.

Data were collected in four segments according to the mass ranges 50–300, 290–600, 590–900, and 890–1500. We used an AB Sciex QTrap 6500 mass spectrometer to develop the assay.

## Results

### Physiological changes of IL7 in response to cold stress

**Fig 1:**
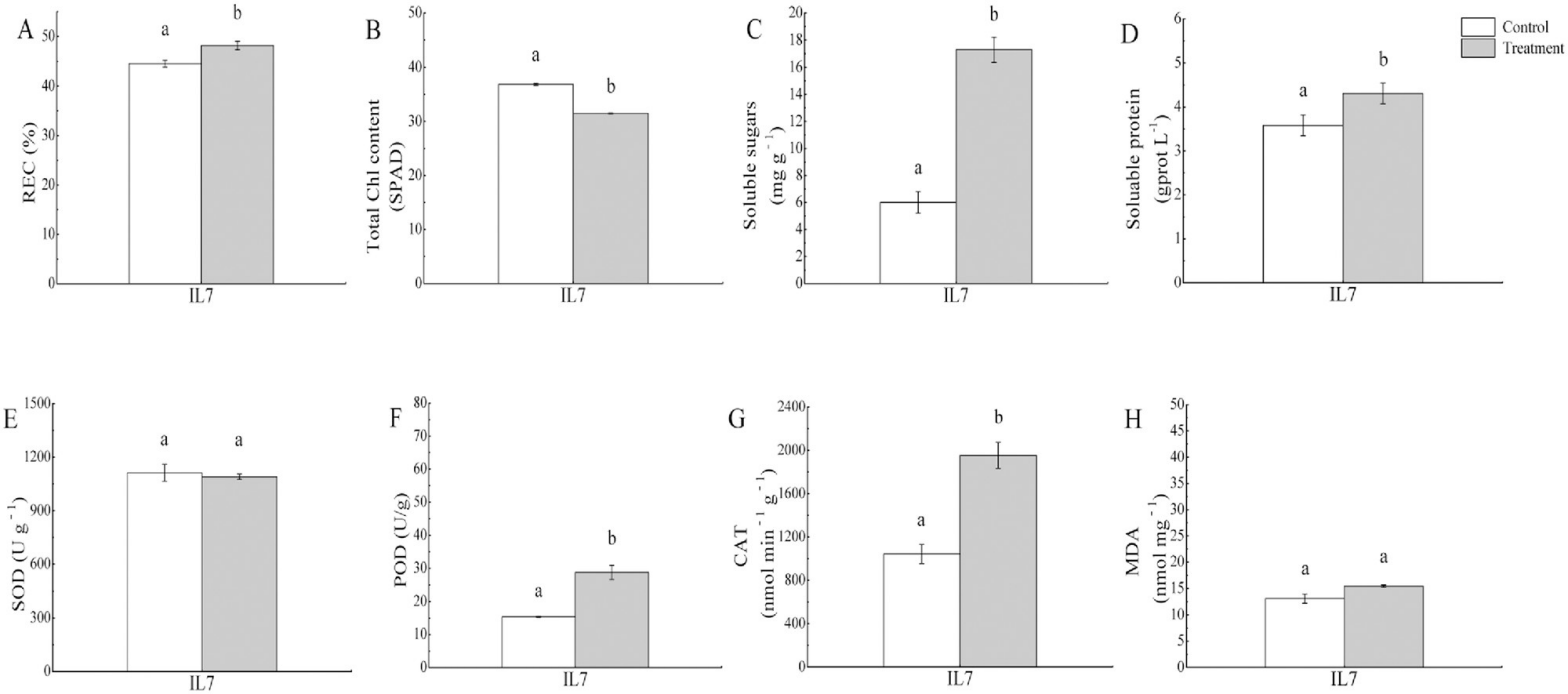
Physiological changes of IL7 under control and cold conditions. A, REC; B, SPAD index; C, Soluble sugar content; D, Soluble protein content; E, SOD; F, POD, G, CAT; H, MDA. Error bars denote standard error of the mean. Bars with the same small letters do not statistically differ by the Duncan’s multiple range test at *P* < 0.05.

The REC, SS content, soluble protein content, POD, and CAT activity in cold-treated IL7 showed a significant increase at 24 h compared with the control, and there was a significant decrease in the SPAD index of cold-treated IL7 relative to the control (Fig 1A–1D, 1F and 1G). However, there was no significant change in SOD activity and MDA content in IL7 (Fig 1E and 1H). In particular, the SS content in the leaves of *C*. *maxima* increased 1.88-fold in fighting the cold stress. These results strongly suggested that the cold-stressed IL7 seedlings actively mounted a stress response after they were exposed to 5 °C.

### Transcriptome analysis of IL7 under cold stress

To further elucidate the molecular basis for cold responses in IL7, a transcriptomic analysis was performed using RNA-seq with a paired-end sequencing strategy on an Illumina HiSeq 2500 platform. Six cDNA libraries representing the control (Sample_IL7C1, Sample_IL7C2, Sample_IL7C3) and, low temperature-treated (Sample_IL7T1, Sample_IL7T2, Sample_IL7T3) treatments samples were constructed. A The full-scale sequencing analysis from of six samples is shown in Table 1. A total of 47.35 to 48.06 million (M) uniquely mapped clean reads were used for the subsequent analysis analyses. A total of 41.53 Gb of clean data were obtained, with an average of 6.92 Gb per library. The percentages of Q30 base was were greater than 94%, and the average GC content was 45.81%. The sample_IL7s were mapped to the *C*. *maxima* genome assembly. The mapped reads in six sample_IL7s were relatively high in the range of 98.40%–98.92%.

**Table 1:** Summary of sequencing data of IL7.

Sample	Clean reads (M)	Clean bases(G)	Q30 (%)	GC (%)	Mapped reads
**IL7C1**	47.35M	6.88G	94.30%	45.96%	98.92%
**IL7C2**	47.87M	6.95G	94.06%	45.95%	98.40%
**IL7C3**	48.06M	6.96G	94.09%	45.81%	98.79%
**IL7T1**	47.49M	6.88G	94.39%	45.59%	98.49%
**IL7T2**	47.76M	6.91G	94.59%	45.71%	98.58%
**IL7T3**	47.90M	6.95G	94.61%	45.82%	98.56%

### Identification of DEGs in response to cold stress

We identified genes expressed in the control and cold-treated leaves of IL7 and found the FPKM value of most genes to be >1 (S1 Fig). The high similarity between the biological replicates of both the control and cold-treated samples indicated that the RNA-seq results were consistent. A correlation analysis of samples also supported consistency (r > 0.99) (S2 Fig). In order to obtain reliable gene expression profiles, reads with a log_2_ |fold change| > 1 and FKPM values > 10 were selected to annotate the DEGs. A total of 5,553 DEGs with 2,871 up-regulated and 2,682 down-regulated genes between the control and cold treated IL7 samples were detected. In addition, 208 up-regulated genes and 44 down-regulated genes had a log_2_ |fold change| > 4.

**Fig 2:**
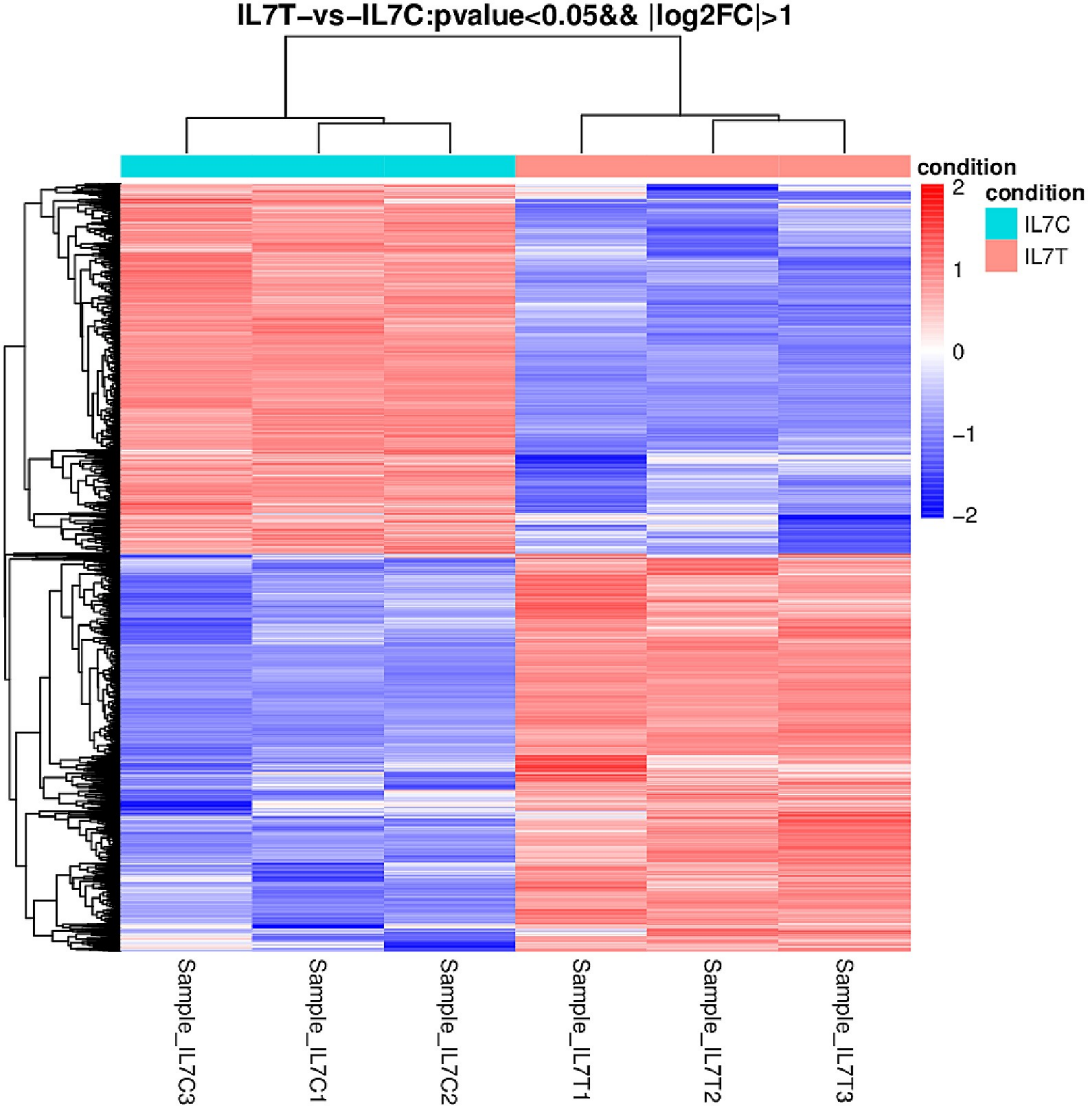
Heat map of IL7T vs IL7C (*P* < 0.05, log2 |Fold Change| > 1). The deepening color in the heat map indicates an increase in the number of gene transcripts at that growth point.

The transcript abundance of all DEGs after cold stress treatment is shown by a hierarchical heat map (Fig 2). The results also showed a series of changes in gene expression in IL7 under cold stress.

### Analysis of transcription factors

As critical regulators, transcription factors (TFs) play key roles in the regulation of gene expression under cold stress in plants. Our RNA-seq results showed that 354 DEGs (221 up-regulated and 133 down-regulated genes) were identified and assigned to various TFs. Among these DEGs, 15 TF genes (two *bHLH*s, seven *ERFs*, four *WRKY*s, and two *MYB*s) were significantly up-regulated by more than 20-fold during cold treatment. Interestingly, most of the predicted *bHLH93* (LOC111476397) and *ERF1B* (LOC111499095) genes showed dramatic levels of activation, as they were up-regulated by 138- and 109-fold, respectively. A total of 252 DEGs were comprised of *AP2/ERF*, *bHLH*, *MYB*, *WRKY*, *HSF*, and *GATA* TFs (Table 2). These genes were the most frequently identified as being up or down-regulated, implying that they might regulate resistance to cold stress. Notably, the large number of AP2/EFR family members indicated that different members may play specific roles in the response to cold stress. These results suggested that these TF families could be involved in the regulation of cold stress.

**Table 2:** Major transcription factor in DEGs.

Number	TF family	Up-genes	Down-genes
**1**	AP2/ERF	60	19
**2**	WRKY	32	7
**3**	BHLH	23	31
**4**	MYB	29	19
**5**	HSF	16	1
**6**	GATA	8	2

### GO and KEGG enrichment results

**Fig 3:**
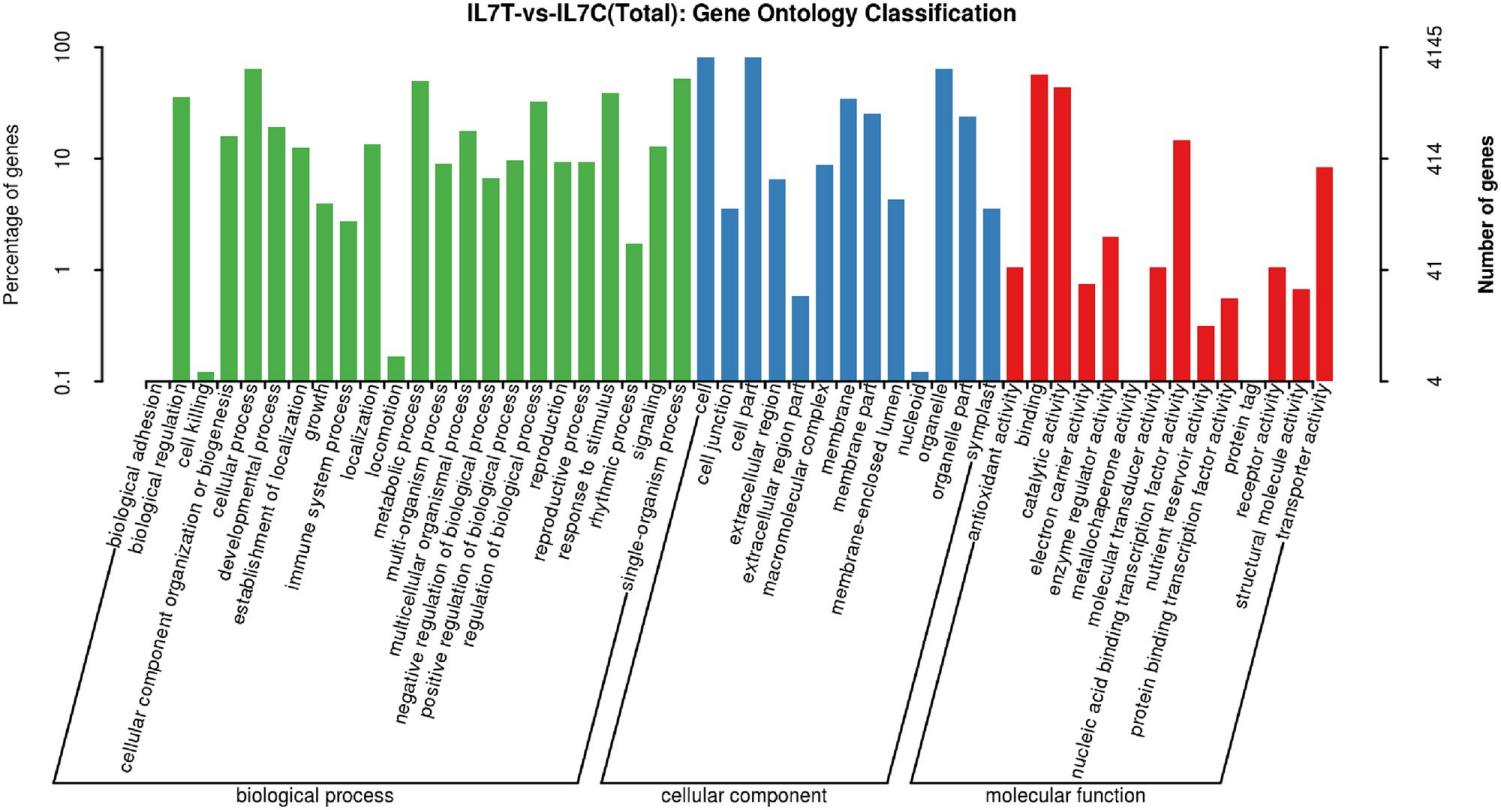
Functional classification of *C*. *maxima* unigenes by using Gene Ontology (GO).

To investigate the functions of the DEGs detected in the IL7 response to cold stress, we analyzed the DEGs by GO term enrichment. GO analysis showed that the DEGs were categorized into functional groups consisting of 1,963 GO terms in biological processes, 405 GO terms in cellular components, and 1,186 GO terms in molecular function subcategories in the comparison between treated and control IL7 samples. The biological process category represented 23 functional subcategories, out of which metabolic process, response to stimulus, and regulation of biological process were predominantly enriched under cold stress (Fig 3). The cellular component and molecular function categories comprise 13 and 14 functional categories, respectively (Fig 3). In the cellular component category, cell, cell part, and organelle were the most highly represented groups. In the molecular function category, binding, catalytic activity, and nucleic acid binding transcription factor activity were the three major groups associated with the *C*. *maxima* unigenes (Fig 3).

**Fig 4:**
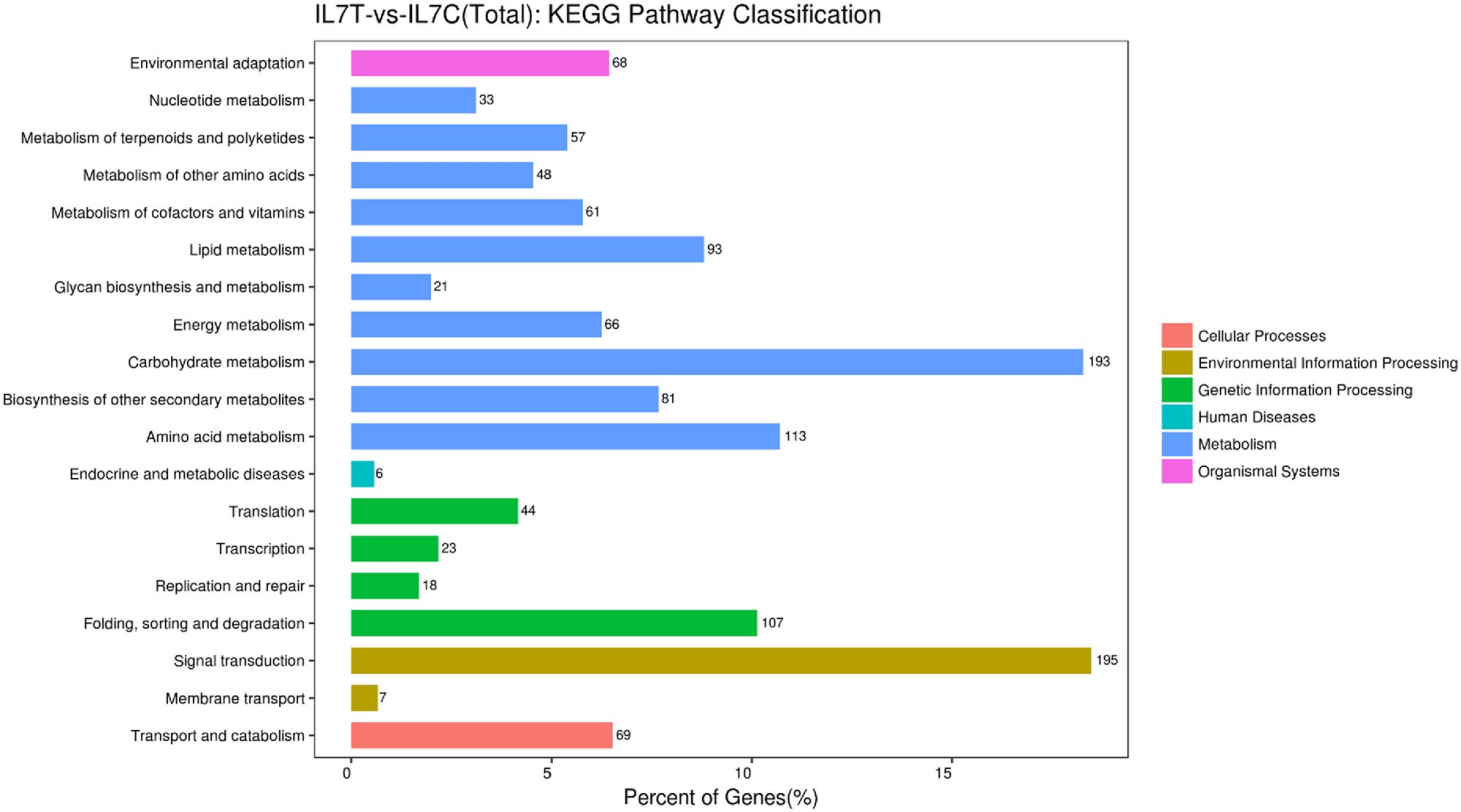
Functional classification of unigenes by using KEGG terms.

**Fig 5:**
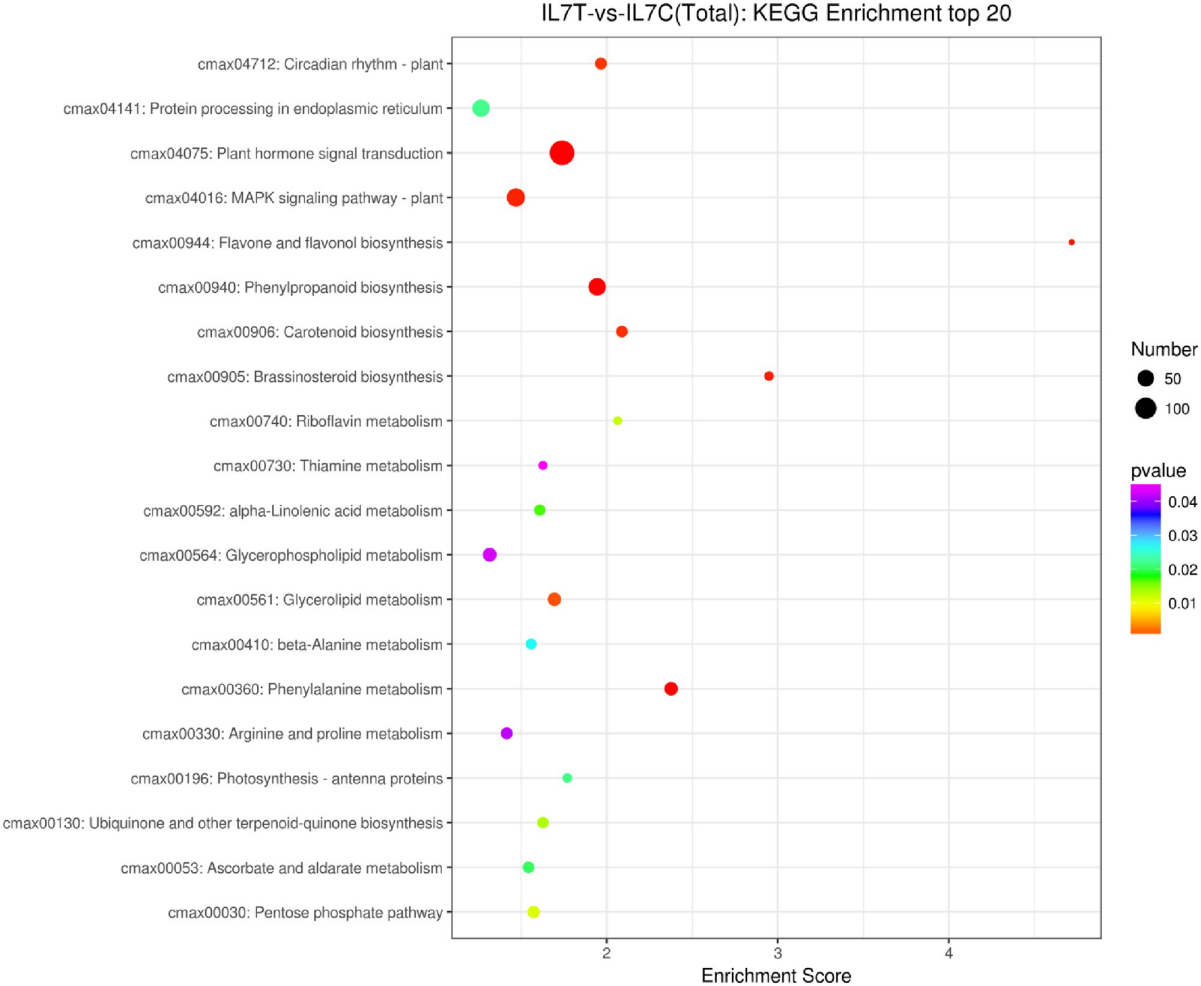
The top 20 KEGG pathways.

KEGG pathway classification assigned 1,529 unigenes were enriched 120 pathways that are involved in metabolism, genetic information, organismal systems, environmental information processing, cellular processes, and human diseases (Fig 4). The most abundant term was signal transduction, followed by the carbohydrate metabolism and amino acid metabolism. The top 20 pathways are shown in Fig 5. The first prominent pathways of significance were plant hormone signal transduction (Fig 5) phenylpropanoid biosynthesis and phenylalanine metabolism, suggesting that these pathways may be closely related to cold stress in IL7.

**Fig 6:**
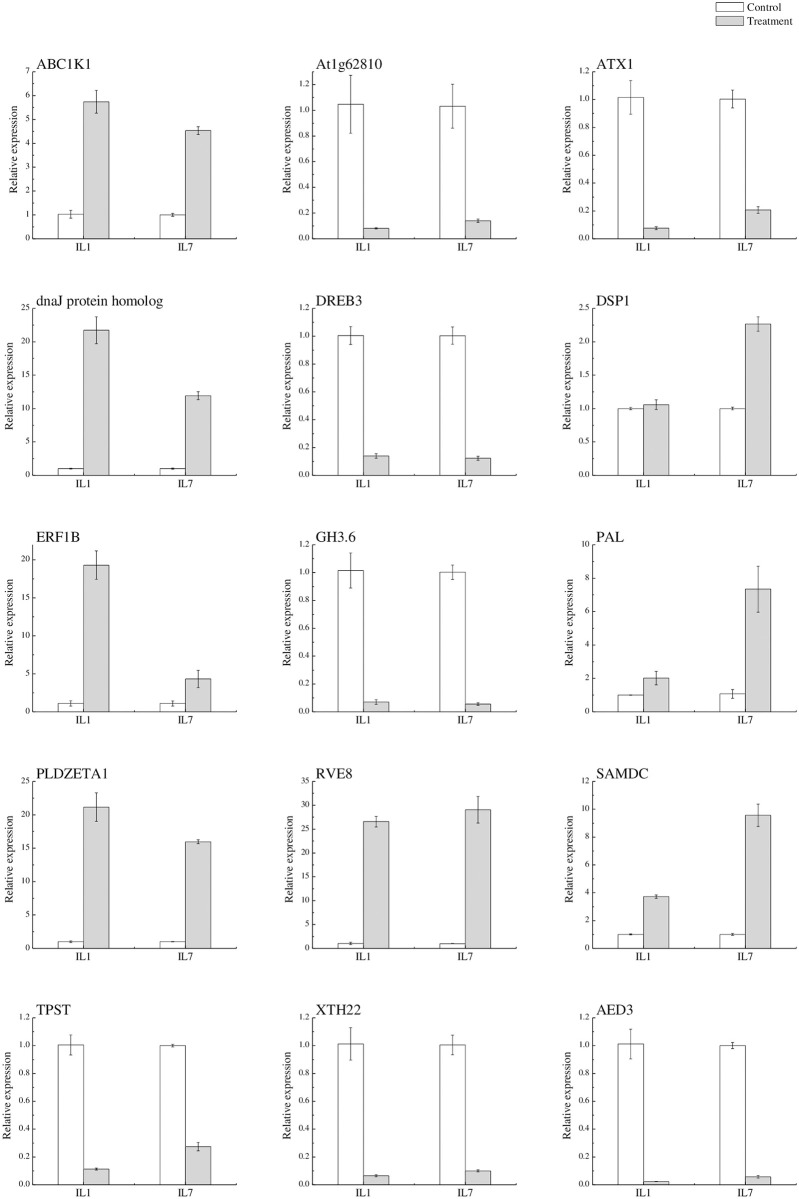
The quantitative RT-PCR results from IL7. Relative quantification was obtained through 2^-ΔΔCT^ method using Actin reference gene. Data represent the average from three biological replicates and the error bars indicate the standard deviation (± SD).

To verify the RNA-seq data, 15 genes were selected for qPCR validation, including *DREB3*, *ERF1B*, and *GH3*.*6*, which have already been reported to be related to cold responses. The results of the RNA-seq and qPCR analyses were similar (Fig 6). The data demonstrated that there was a strong positive correlation (R2 = 0.963) between the RNA-seq data and qPCR results.

### Differential expression of plant hormone signal transduction

Hormones are signaling molecules that regulate gene expression under cold stress. A total of 148 DEGs were associated with auxin (AUX/indole-3-acetic acid) (IAA), cytokinin (CK), gibberellin (GA), abscisic acid (ABA), ethylene (ET), brassinosteroid (BR), jasmonic acid (JA), and salicylic acid (SA)-mediated pathways (S2 Table).

In detail, 53 genes related to auxin were differentially expressed, including 20 AUX/indole-3-acetic acids (*Aux/IAA*), 21 small Auxin-Up RNAs (*SAUR*s), six *AUX1*s, four Gretchen Hagen 3s (*GH3*s), and one auxin response factor (*ARF*) involved in auxin-induced signal transduction pathways. Most of the gene expression were down-regulated, indicating that the auxin signaling pathway was inhibited. Thirteen DEGs were significantly enriched in cytokinin-mediated pathway; one of the five up-regulated *A-ARR* genes displayed the highest level of expression with a fold change of 39.15. Six DEGs (five up-regulated and one down-regulated) were involved in GA-mediated pathways; the *GIDI* and *GELLA* genes showed prominent up-regulation with 16.34-fold and 7.17-fold changes, respectively. Up-regulated expression of these genes suggested cytokinin-mediated pathway was activted Twenty-three DEGs were related to ABA hormone-mediated signaling; the *PP2C* gene (LOC111467353) showed up-regulation of 16.1-fold, suggesting that it might play a vital role against cold stress. Moreover, 11 up-regulated and two down-regulated DEGs were found to be regulated via ET. An *ERF1/2* gene associated with the ET-signaling pathway was the most highly expressed and exhibited a 109.0-fold change in expression. Fifteen DEGs were associated with BR-mediated pathways (four up-regulated and 11 down-regulated); *TCH4*, which exhibited a 35.83-fold change in expression was highly induced under cold stress. Furthermore, ten DEGs (nine up-regulated and one down-regulated) were found to be linked with JA-mediated pathways; *JAR1*, *JAZ*, and *MYC2* were induced by more than three-fold. Three *NPR1* genes (two up-regulated and one down-regulated) and five *TGA* genes (three up-regulated and two down-regulated) were found to be linked with SA-mediated pathways; one *NPR1* gene exhibited a 3.02-fold change in expression and one *TGA* gene displaying a 3.11-fold change in expression were highly expressed in IL7 under cold stress.

### Differential expression of genes related to phenylpropanoid biosynthesis

The phenylpropanoid biosynthesis pathway is activated under abiotic stresses including drought, high/low temperature, heavy metal toxicity, salinity, and ultraviolet radiation; thus, this pathway has the potential to scavenge excess ROS and maintain the cellular redox status [30,31].

**Fig 7:**
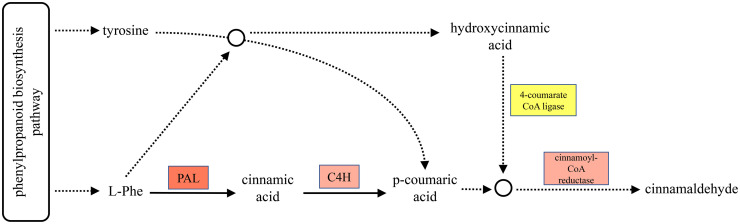
Phenylpropanoid biosynthesis pathway.

In the present study, KEGG enrichment indicated the presence of 59 DEGs (31 up-regulated and 18 down-regulated) and demonstrated that phenylpropanoid biosynthesis (Fig 7) played important roles in the resistance of *C*. *maxima* to cold stress. The fist enzyme in the phenylpropanoid biosynthesis pathway is phenylalanine ammonia lyase (PAL), which catalyzes the conversion of L-Phe into cinnamic acid [32]. In all of the plants studied, PAL is present in multiple isoforms; thus, this enzyme must belong to a multi-gene family [33]. Likewise, nine *PAL* isoform genes were up-regulated in *C*. *maxima* under cold stress. The trans-cinnamate 4-monooxygenase-like (C4H) enzyme, is a member of the cytochrome P450 monooxygenase superfamily and is also the second key enzyme in the phenylpropanoid biosynthesis pathway, which catalyzes the hydroxylation of trans-cinnamic acid to p-coumaric acid [34]. In our study, only two *C4H*s of *C*. *maxima* were up-regulated with a log_2_ |fold change| = 7.8 and log2 |fold change| = 4.1 under cold treatment. The third enzyme in the phenylpropanoid biosynthesis pathway is 4-coumarate CoA ligase, which may play a central role in regulating the entry of hydroxycinnamic acid into the subsequent biosynthetic pathway. Under cold treatment in *C*. *maxima*, three 4-coumarate CoA ligases were up-regulated and four down-regulated. In addition, cinnamoyl-CoA reductase is the most important and final step in the synthesis of cinnamaldehyde, and two cinnamoyl-CoA reductases were more highly expressed in the leaves of *C*. *maxima* under cold stress.

### Detection and profile analysis of metabolites under cold stress

**Fig 8:**
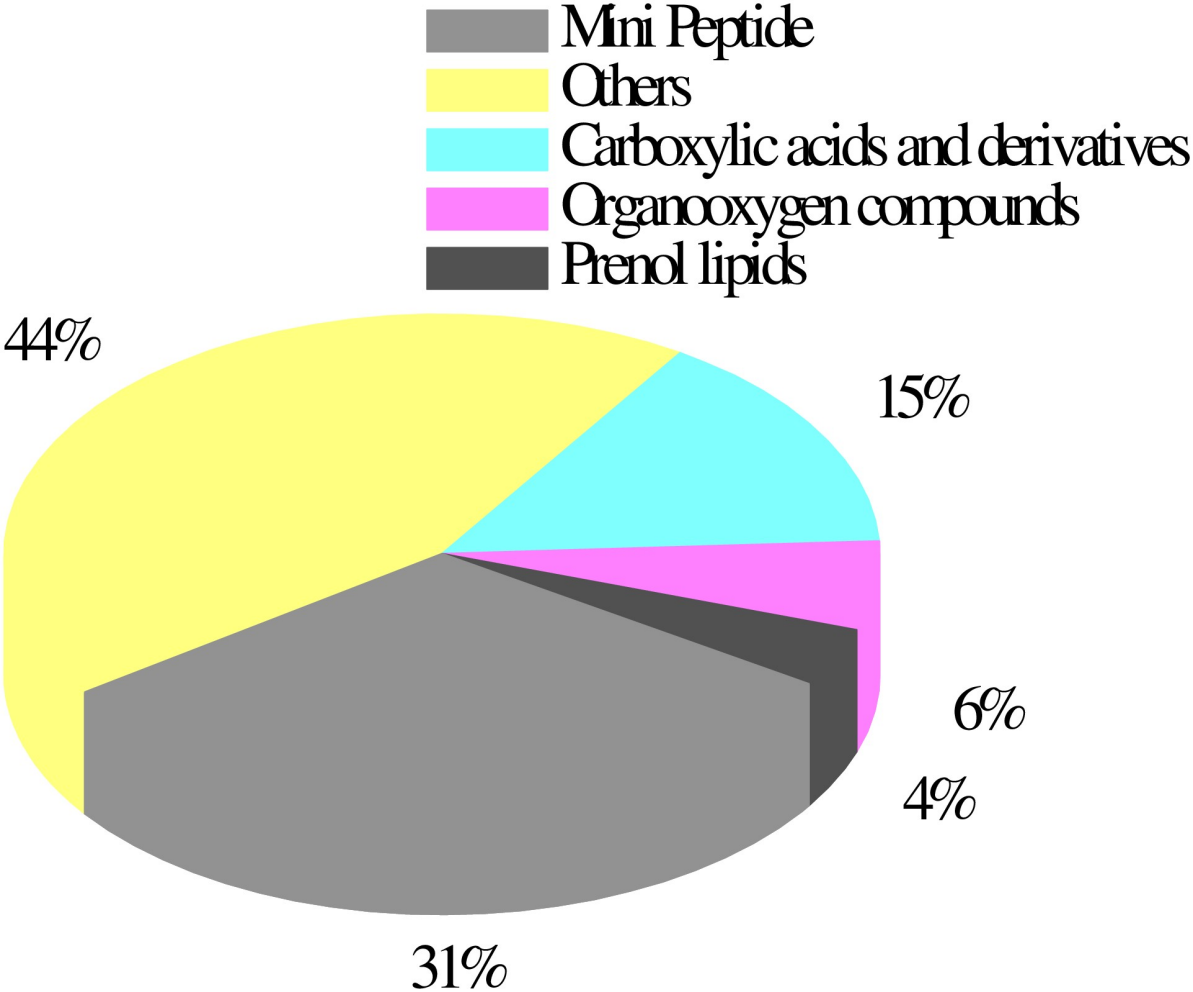
Differentially accumulated metabolites in the *C*. *maxima* after cold stress mainly belonged to four classes.

To understand the metabolome changes associated with IL7 under cold stress, an untargeted metabolite analysis was performed on young leaves using UHPLC quantitative time-of-flight MS. The samples consisted of two groups: IL7C (control) and IL7T (treatment). As seen from the results of the orthogonal partial least squares discriminant analysis score map, the differences in the two groups of samples were very significant and all were within the 95% confidence interval (S3 and S4 Figs). A total of 114 metabolites showed differential accumulation (using VIP ≥ 1 and T-test *P* < 0.05,) in IL7, with 34 increased and 80 decreased metabolites, including 20 different classes of substances (S2 Table). Among increased metabolites, carboxylic acids and derivatives, organooxygen compounds, fatty acyls, and cinnamic acids and derivatives were themoste prominent in *C*. *maxima* (Fig 8). More increase of carboxylic acids and derivatives, such as L-phenylalanine and L-tyrosine, were important members of amino acids metabolism (S2 Table).

## Discussion

The response to cold stress in plants is a process by which plants adapt to the environment through gene expression and metabolism [35–38]. In this study, the physiological changes in *C*. *maxima* after cold treatment revealed considerable variation related to cold stress. Significant variations in gene expression and metabolite levels after cold stress were detected using RNA-Seq and LC-MS analysis, respectively.

### Transcription factor in response to cold stress

The transcriptional regulation of plant genes is an important step in the response to cold stress [39–42]. Thus, it is important to elucidate the complex regulatory mechanisms behind gene expression in plants to reveal the molecular basis of plant tolerance to low temperatures [43–46]. Members of several TF families, including AP2/ERF [47–49], WRKY [50,51], and MYB [14,15,52,53] play crucial roles in diverse stress responses. The AP2/ERF family, which is divided into the AP2/ERF, RAV and DREB subfamilies, plays important roles in multiple stress responses [54,55]. Since the first AP2/ERF TF was cloned from *A*. *Thaliana* [56], the biological functions of some *AP2/ERF* genes have been identified. For instance, overexpression of the rice *OsDRE1F* gene can increase tolerance to salt, drought, and low temperatures in both *Arabidopsis* and rice [57], and ectopic overexpression of a citrus ERF1 gene enhances cold tolerance in tobacco [58], while the birch *BpERF13* gene demonstrates a positive regulation of cold stress by upregulating a *CBF* gene and reducing the number of reactive oxygen species (ROS) [59]. In the present study, *AP2/ERF* TFs were represented by 53 up-regulated and 18 down-regulated genes under cold stress. These results are in conformity with the previous reports on low temperature stress regulation due to presence of AP2/ERF family [60–62]. WRKY TFs represent a valuable family in the resistance to abiotic stresses such as cold, heat, drought, and salt. For example, a total of 17 members of this family were induced by chilling in japonica rice [60]. And a set of *WRKY* genes were also found to be chilling-regulated in indica rice, of which 12 were commonly induced, 22 were exclusively up-regulated in cold susceptible variety and 18 were exclusively up-regulated in cold tolerant variety genotype [63]. Moreover, the roles of *VaWRKY12* from *Vitis amurensis* in cold stress tolerance were confirmed through its ectopic overexpression in *Arabidopsis* and grapevine [64]. A *VbWRKY32* in the seedling stage of *Verbena bonariensis* upregulates the transcriptional level of cold response genes, which increases the antioxidant enzyme activities and the contents of osmotic adjustment substance, thereby improving the survival ability under cold stress [65]. Taken together, 33 *WRKY* DEGs of *C*. *maxima* may contribute to the further study of cold tolerance mechanisms. In addition, members of the MYB family have been shown to be pivotal factors in regulating responses to abiotic stress. Some *MYB* TFs are involved in both cold and chilling stresses. For example, the expression of *LcMYB4* in *Leymus chinensis* was rapidly induced by cold treatment, and positively modulates chilling and freezing tolerance in *Arabidopsis* [66]. However, *VcMYB4a* of *Vaccinium corymbosum* transcript levels gradually decreased during the cold treatment [67]. Moreover, *SlMYB102* in tomato enhances antioxidant enzyme activity and promotes the expression of cold resistance under low temperature stress conditions [52]. Similar to previous studies, the expressions of *MYB* genes in *C*. *maxima* were induced with the upregulated and downregulated pattern during cold stress. In the future, the mechanism of cold tolerance of these genes needs to be further studied. Recently, some studies have shown that *AP2/ERF*, *WRKY*, and *MYB* TFs are also involved in the expression of genes related to the phenylpropanoid pathway [68–70]. In our results, major TF families such as *AP2/ERF*, *MYB*, *WRKY*, and *MYB* were represented by a large number of DEGs and therefore have a significant influence on the resistance to cold stress in *C*. *maxima*. Thus, these TFs are likely to be involved in complex mechanisms associated with temperature regulation.

### Plant hormone signaling involved in cold stress

Plant hormones play important roles in mediating development processes and defense response to various biotic and abiotic stresses [71,72]. Many genes indeed displayed altered expression levels in several hormone-mediated branches, such as ABA, IAA, CK, GA, ET, BR, JA, and SA. Obviously, most of the genes in auxin signaling pathway in *C*. *maxima* leaves exposed to cold stress were inhibited, which generally are obligated to cell elongation, division, cycle, and growth [13,73]. Auxin is an endogenous small molecule with an incredibly large impact on plant growth and and response to stress in concert with other hormonal pathways [74–76]. The Auxin response factor (ARF), Gretchen Hagen 3 (GH3), and Auxin/indole-3-acetic acid (Aux/IAA), small Auxin-Up RNA (SAUR) gene families, are key components of the auxin signaling pathway, and function as regulators of plant growth and response to stress [77,78]. More recently, auxin signal in strawberry (*Fragaria*×*ananassa*) was severely blocked after low temperature treatment, indicating that auxin signal plays a very important role in cold stress [13]. This conclusion is consistent with our finding in *C*. *maxima*. CK is strongly cross-talks with auxins in plant developmental processes [2]. In *Arabidopsis*, CKs initiate signalling utilising *Arabidopsis* histidine kinase (AHK)2, AHK3 and AHK4, which act as CK receptors. Upon CK binding, the receptors transfer the CK signal via *Arabidopsis* histidine phosphotransfer proteins (AHPs) to nuclear localised *Arabidopsis* response regulators (ARRs), TFs that regulate expression of CK target genes [79]. In *Arabidopsis*, there are 23 ARR proteins, and transcript levels of A-type ARR5, ARR7 and ARR15 were induced in response to cold in a AHK-dependent manner [80,81]. Some A-type and B-type ARRs in *C*. *maxima* were induced expression, sugessting that CK signaling was activeted after cold stress. Quite on the contrary, as opposed to GAs, BRs are thought to positively control cold stress responses, because it has been shown that BR application increases cold tolerance of many plant species, including chilling-sensitive crops such as maize and *Cucumis sativus* (cucumber) [81–84]. Previous studies showed that GA and JA can cooperatively regulate diverse aspects of plant growth, development, and defense through DELLA and JAZ proteins [85,86], and JA prioritizes defense over growth by interfering with GA signaling cascade [48]. ABA, a central regulator of cold stress signalling with emerging roles in the CBF-dependent pathway [2]. Cold treatment of pumpkin activated hormone signaling pathway, which was confirmed by some differential expression of genes.

### Carboxylic acids and derivatives metabolites in response to cold stress

In addition, the associated accumulation of specific amino acids has been correlated with increased tolerance to stressful environmental conditions [87]. Phenylalanine and tyrosine have different functions in stress resistance and are central to plant metabolism [88]. The increase in amino acids in *A*. *thalian*a, tomato, and other plants under during stress acclimation indicate that the accumulation of amino acids is related to the degradation of proteins [13,89,90]. It has been reported that the exogenous application of phenylalanine can promote chilling tolerance in tomato by activating ROS-scavenging systems; an increase in the abundance of the phenylalanine is also induced by low temperature [91]. L-phenylalanine (L-phe) is produced from phenylpyruvic acid-mediated amination by aspartate aminotransferase [92]. Detection of metabolism showed that the accumulation of L-Phe and tyrosine provided important materials to the phenylpropanoid biosynthesis pathway. Moreover, the pathway is also involved in cold tolerance in winter rapeseed and grape [10,18]. The current results indicated that the amino acid metabolism in *C*. *maxima* was induced and helped in the resistance to a cold environment.

## Conclusions

Cold stress has an important influence on plant growth and development, leading to physiological, biochemical, metabolic, and molecular changes. In the present study, we comprehensively analyzed the changes in the transcriptome and metabolome from *C*. *maxima* leaves during cold stress. The results revealed that cold stress broadly activated the pathways for plant hormone signal transduction, phenylpropanoid biosynthesis. Important genes and metabolic processes in these pathways during cold stress could provide valuable information for further functional characterization in future studies.

## Supporting information

S1 Fig The FPKM expression distribution map of IL7T vs IL7C. (TIF)

S2 Fig Correlation coefficient heat map between samples of IL7. (TIF)

S3 Fig The Score scatter plot of OPLS-DA for group IL7T vs IL7C. (TIF)

S4 Fig Permutation test of OPLS-DA model for group IL7T vs IL7C. (TIF)

S1 Table Information of gene specific primers for qRT-PCR. (DOCX)

S2 Table DEGs involving in plant hormone signal transduction. (DOCX)
