# Supplementary figures and images for: Integrating transcriptome and metabolome analyses of the response to cold stress in pumpkin (Cucurbita maxima)
Source: PLoS One. 2021 May 6;16(5):e0249108. doi: 10.1371/journal.pone.0249108 (PMC8101736; doi:10.1371/journal.pone.0249108)

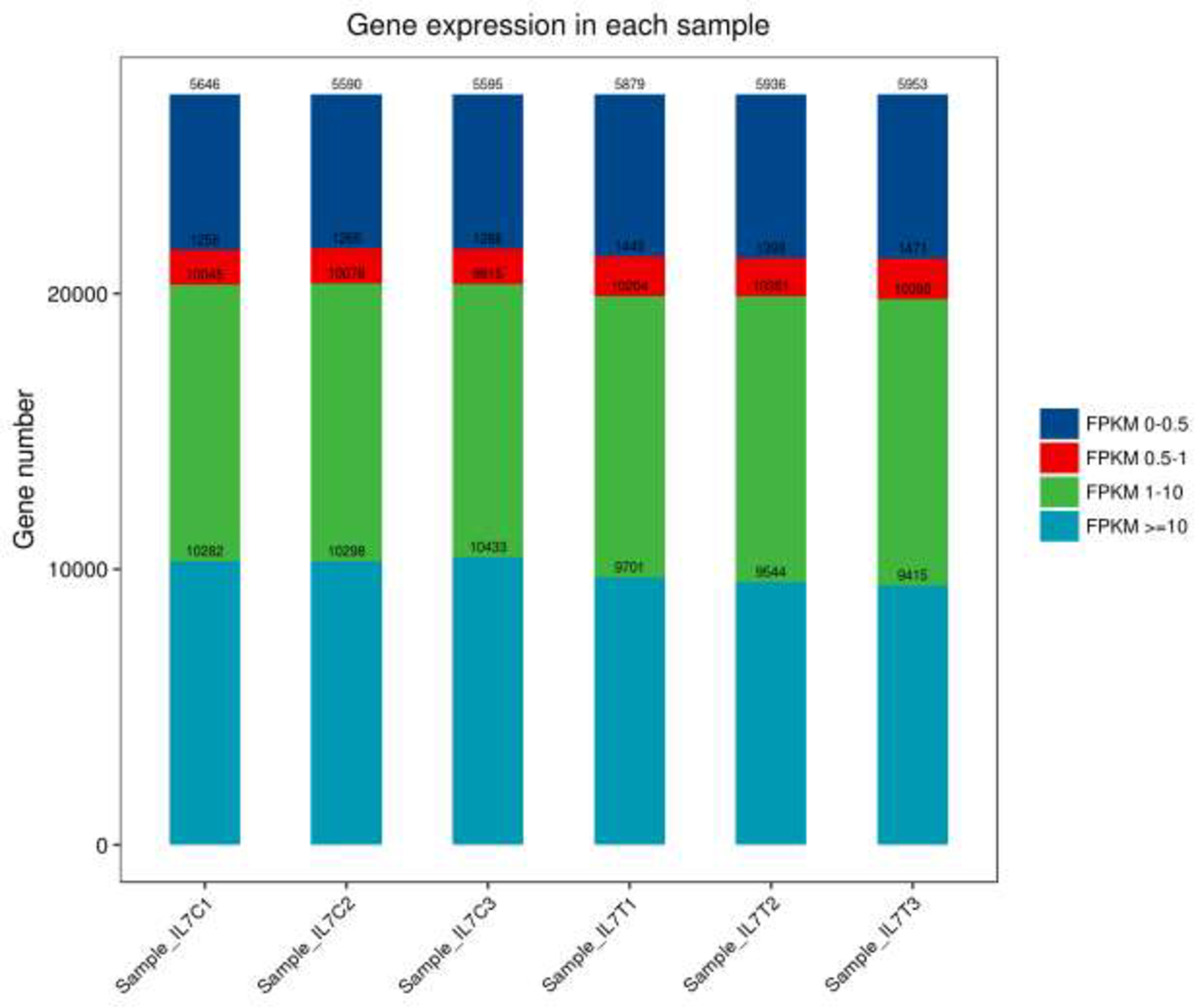

Supplement: S1 Fig — (TIF) [file pone.0249108.s001.tif]

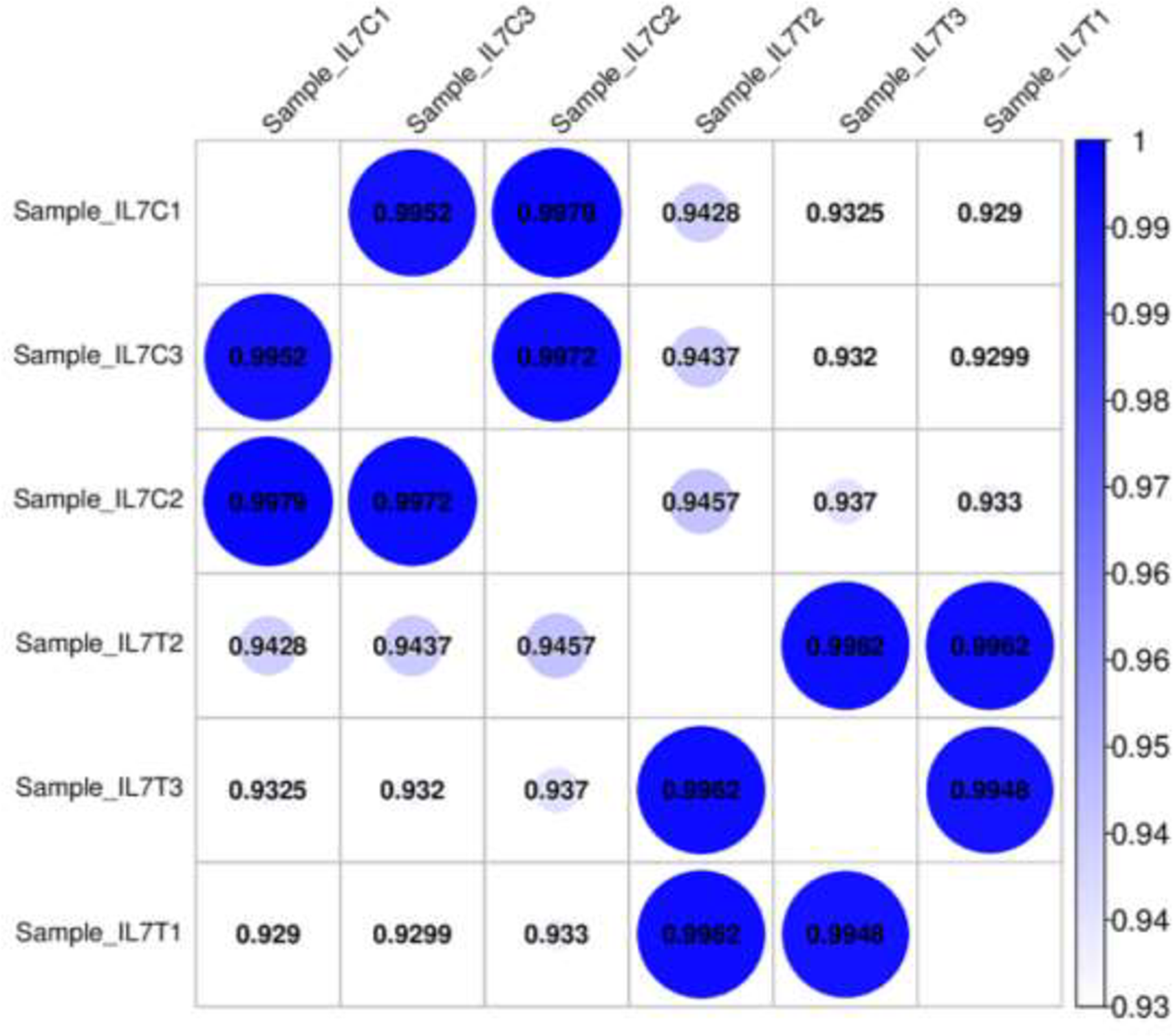

Supplement: S2 Fig — (TIF) [file pone.0249108.s002.tif]

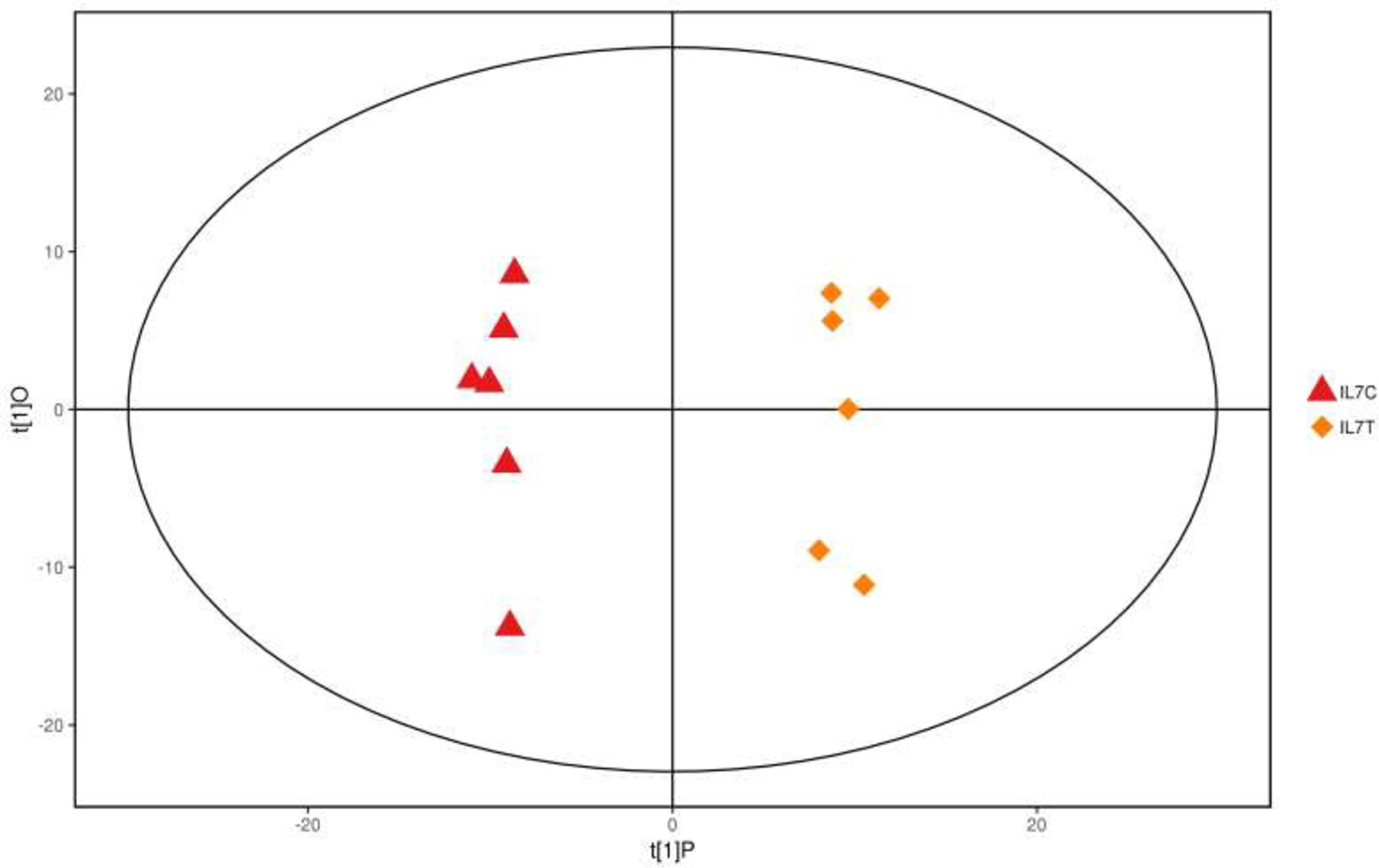

Supplement: S3 Fig — (TIF) [file pone.0249108.s003.tif]

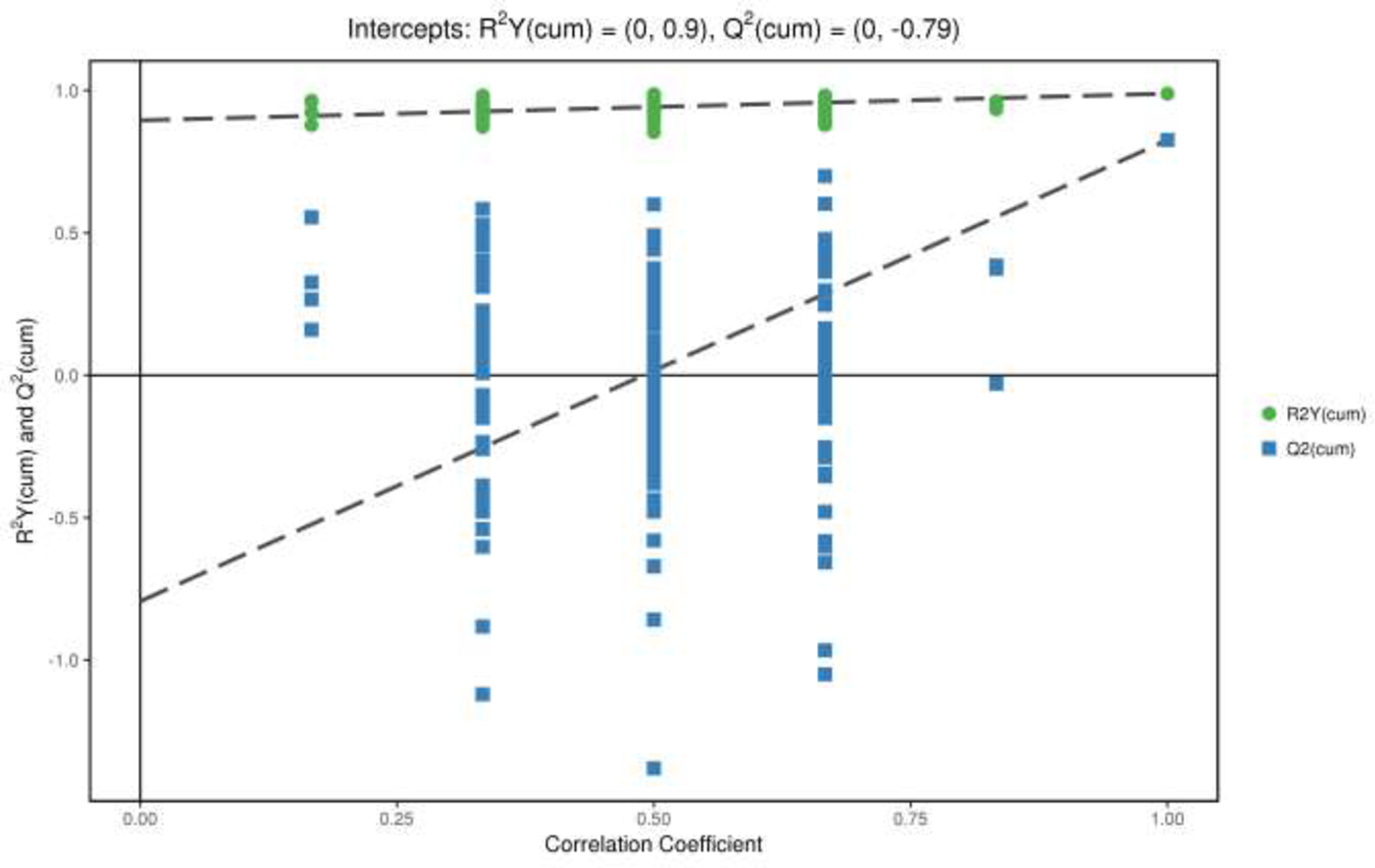

Supplement: S4 Fig — (TIF) [file pone.0249108.s004.tif]
