# Supplementary material for: Integrating transcriptome and metabolome analyses of the response to cold stress in pumpkin (Cucurbita maxima)
Source: PLoS One. 2021 May 6;16(5):e0249108. doi: 10.1371/journal.pone.0249108 (PMC8101736; doi:10.1371/journal.pone.0249108)
Supplement: S1 Table — (DOCX) [file pone.0249108.s005.docx]

**S1Table. Information of gene specific primers for qRT-PCR.**

| **No.** | **Gene** | **Gene ID** | **Forward primer** | **Reverse primer** |
| --- | --- | --- | --- | --- |
| **1** | AED3 | LOC111492140 | TAGCGTCGATTGCTTATTCAAC | GATCCCAAGTGGAGAGTGT |
| **2** | ABC1K1 | LOC111492323 | GCTTTGTCGGAAGGGATG | ACGAAGGTCCCAAATTACAC |
| **3** | RVE8 | LOC111482132 | GCTCCACAAGTTCTCATTTCAC | AGCATGATAAGGCTGAATCG |
| **4** | XTH22 | LOC111497152 | AGAGGGAATCATATTCTCGGT | GTCTCATGGGTTGCTTTGTC |
| **5** | ATX1 | LOC111481086 | CTTACTGCTTGCCGACGA | AGGAGGCTTGTATTCAACATT |
| **6** | At1g62810 | LOC111491661 | TTTCTCGGGCCAAAGCTC | GACGGGTCCATGTAAGGCA |
| **7** | GH3.6 | LOC111476851 | TCTCACAAGCTCGGGAAC | CCATGAATTGGCTCATCACT |
| **8** | PLDZETA1 | LOC111493911 | GTAAGAGAACACGGTCCGAA | CTGCACCAATGACATGCAA |
| **9** | DSP1 | LOC111469661 | CGAGAGCTATAAGGAGCC | ATTTCTATCGTCAAGGACAACT |
| **10** | TPST | LOC111468614 | AGAACAGGAGGGCGATCATA | CTAGCAATCTGCACTTGGC |
| **11** | ERF1B | LOC111474018 | AATGAGAATGATTCCGAGGAAA | CCCAATTCCCTTCTCGGAC |
| **12** | DnaJ protein homolog | LOC111485072 | AGTTGTTCAAGAGAAGAAGGTT | TCTGCTTCTCCAGGGAATGTA |
| **13** | SAMDC | LOC111492585 | AATATGTGACTTCGAGTTCGAC | TCTGGAGTTACATGGATGGTAG |
| **14** | PAL | LOC111471134 | GGAACCATCACTGCTTCG | CCGCCTTAGAGTTGGGTC |
| **15** | DREB3 | LOC111480318 | TAGCACAACAATGTCCCAAAC | CTTGCTGGAATCTCGAACG |
| **16** | Actin | LOC111487712 | AGCCATCTCTCATCGGTAT | CATGGTTGAACCACCACTG |
