# Supplementary material for: Integrating transcriptome and metabolome analyses of the response to cold stress in pumpkin (Cucurbita maxima)
Source: PLoS One. 2021 May 6;16(5):e0249108. doi: 10.1371/journal.pone.0249108 (PMC8101736; doi:10.1371/journal.pone.0249108)
Supplement: S2 Table — (DOCX) [file pone.0249108.s006.docx]

**S2 Table. DEGs involving in plant hormone signal transduction.**

| **Plant hormone signing transduction** |  | **Gene ID** | **FC** | **Log2FC** |
| --- | --- | --- | --- | --- |
| **Auxin (IAA)** | ***AUX1*** | LOC111466192 | 0.19 | -2.40 |
|  |  | LOC111478219 | 0.04 | -4.64 |
|  |  | LOC111469798 | 0.16 | -2.65 |
|  |  | LOC111486236 | 0.33 | -1.59 |
|  |  | LOC111468472 | 0.25 | -2.01 |
|  |  | LOC111486960 | 0.38 | -1.38 |
|  | ***T1R1*** | LOC111478106 | 2.09 | 1.06 |
|  | ***AUX/IAA*** | LOC111492883 | 0.17 | -2.56 |
|  |  | LOC111499386 | 0.46 | -1.13 |
|  |  | LOC111481003 | 0.33 | -1.58 |
|  |  | LOC111479547 | 0.08 | -3.66 |
|  |  | LOC111490866 | 2.04 | 1.03 |
|  |  | LOC111495511 | 2.16 | 1.11 |
|  |  | LOC111483950 | 0.04 | -4.80 |
|  |  | LOC111498041 | 0.06 | -3.97 |
|  |  | LOC111474863 | 3.40 | 1.77 |
|  |  | LOC111469303 | 0.12 | -3.10 |
|  |  | LOC111494782 | 0.36 | -1.48 |
|  |  | LOC111471496 | 9.59 | 3.26 |
|  |  | LOC111492708 | 0.31 | -1.69 |
|  |  | LOC111488703 | 0.10 | -3.28 |
|  |  | LOC111493783 | 0.35 | -1.54 |
|  |  | LOC111493578 | 0.06 | -4.13 |
|  |  | LOC111498932 | 2.46 | 1.30 |
|  |  | LOC111497647 | 0.46 | -1.11 |
|  |  | LOC111492157 | 0.48 | -1.07 |
|  |  | LOC111472949 | 2.65 | 1.41 |
|  | ***ARF*** | LOC111483404 | 2.81 | 1.49 |
|  | ***GH3*** | LOC111497055 | 0.11 | -3.24 |
|  |  | LOC111478067 | 13.34 | 3.74 |
|  |  | LOC111476851 | 0.11 | -3.25 |
|  |  | LOC111479427 | 0.44 | -1.18 |
|  | ***SAUR*** | LOC111469290 | 0.16 | -2.65 |
|  |  | LOC111499611 | 2.09 | 1.07 |
|  |  | LOC111470869 | 12.96 | 3.70 |
|  |  | LOC111484391 | 0.16 | -2.63 |
|  |  | LOC111484392 | Inf | Inf |
|  |  | LOC111484393 | 0.36 | -1.49 |
|  |  | LOC111468489 | 0.06 | -4.01 |
|  |  | LOC111486306 | 0.14 | -2.87 |
|  |  | LOC111486308 | 0.13 | -2.97 |
|  |  | LOC111486309 | 0.08 | -3.72 |
|  |  | LOC111480219 | 2.95 | 1.56 |
|  |  | LOC111476445 | 4.28 | 2.10 |
|  |  | LOC111484320 | 0.13 | -2.97 |
|  |  | LOC111469701 | 4.30 | 2.10 |
|  |  | LOC111484401 | 0.24 | -2.05 |
|  |  | LOC111484403 | 0.11 | -3.15 |
|  |  | LOC111476547 | 0.13 | -2.95 |
|  |  | LOC111468493 | 0.13 | -2.93 |
|  |  | LOC111468494 | 0.13 | -2.92 |
|  |  | LOC111468495 | 0.22 | -2.17 |
|  |  | LOC111468496 | 3.46 | 1.79 |
| **Cytokinin (CK)** | ***CRE1*** | LOC111497614 | 0.24 | -2.08 |
|  |  | LOC111486406 | 2.05 | 1.03 |
|  | ***AHP*** | LOC111472263 | 2.44 | 1.29 |
|  |  | LOC111492700 | 12.87 | 3.69 |
|  |  | LOC111479501 | 0.39 | -1.38 |
|  | ***B-ARR*** | LOC111467372 | 7.43 | 2.89 |
|  |  | LOC111480378 | 0.19 | -2.37 |
|  |  | LOC111498799 | 2.13 | 1.09 |
|  | ***A-ARR*** | LOC111486903 | 39.15 | 5.29 |
|  |  | LOC111489700 | 6.31 | 2.66 |
|  |  | LOC111485658 | 5.81 | 2.54 |
|  |  | LOC111488059 | 2.58 | 1.37 |
|  |  | LOC111465933 | 3.97 | 1.99 |
| **Gibberellin (GA)** | ***GID1*** | LOC111497844 | 16.34 | 4.03 |
|  |  | LOC111469829 | 2.15 | 1.10 |
|  |  | LOC111488829 | 5.56 | 2.48 |
|  | ***DELLA*** | LOC111472091 | 2.91 | 1.54 |
|  |  | LOC111492824 | 7.17 | 2.84 |
|  | ***TF*** | LOC111481266 | 0.34 | -1.54 |
| **Abscisic acid** | ***PYRPYL*** | LOC111471565 | 0.28 | -1.84 |
|  |  | LOC111466241 | 0.31 | -1.70 |
|  |  | LOC111490972 | 0.35 | -1.53 |
|  |  | LOC111498494 | 2.70 | 1.43 |
|  | ***PP2C*** | LOC111485188 | 2.51 | 1.33 |
|  |  | LOC111489250 | 5.34 | 2.42 |
|  |  | LOC111492731 | 3.02 | 1.59 |
|  |  | LOC111499429 | 4.63 | 2.21 |
|  |  | LOC111467353 | 16.10 | 4.01 |
|  |  | LOC111479918 | 2.39 | 1.26 |
|  |  | LOC111483131 | 9.93 | 3.31 |
|  |  | LOC111493428 | 6.13 | 2.62 |
|  | ***SnRK2*** | LOC111496805 | 2.68 | 1.42 |
|  |  | LOC111499644 | 0.28 | -1.82 |
|  |  | LOC111478475 | 2.44 | 1.29 |
|  |  | LOC111498437 | 0.41 | -1.29 |
|  |  | LOC111499972 | 0.29 | -1.79 |
|  |  | LOC111470368 | 0.34 | -1.54 |
|  | ***ABF*** | LOC111487275 | 0.39 | -1.37 |
|  |  | LOC111491837 | 2.23 | 1.16 |
|  |  | LOC111486047 | 3.06 | 1.61 |
|  |  | LOC111483731 | 7.40 | 2.89 |
|  |  | LOC111487283 | 3.30 | 1.72 |
| **Ethylene** | ***ETR*** | LOC111474519 | 2.44 | 1.29 |
|  |  | LOC111479148 | 2.68 | 1.42 |
|  |  | LOC111497779 | 2.42 | 1.27 |
|  |  | LOC111491724 | 2.58 | 1.37 |
|  |  | LOC111474608 | 2.21 | 1.15 |
|  | ***CTR1*** | LOC111481573 | 0.49 | -1.04 |
|  | ***SIMKK*** | LOC111479957 | 2.43 | 1.28 |
|  |  | LOC111493349 | 2.10 | 1.07 |
|  | ***EBF1/2*** | LOC111476613 | 2.09 | 1.07 |
|  | ***EIN3*** | LOC111479871 | 4.20 | 2.07 |
|  | ***ERF1/2*** | LOC111474018 | 5.23 | 2.39 |
|  |  | LOC111499095 | 109.00 | 6.77 |
|  |  | LOC111480113 | 0.27 | -1.87 |
| **Brassinosteroid** | ***BKI1*** | LOC111476759 | 0.22 | -2.21 |
|  | ***BSK*** | LOC111469792 | 0.42 | -1.27 |
|  |  | LOC111481316 | 0.48 | -1.05 |
|  |  | LOC111495994 | 2.17 | 1.12 |
|  |  | LOC111479887 | 0.40 | -1.33 |
|  |  | LOC111479697 | 0.45 | -1.16 |
|  | ***BIN2*** | LOC111468215 | 0.38 | -1.41 |
|  | ***TCH4*** | LOC111485035 | 35.33 | 5.14 |
|  |  | LOC111497152 | 0.12 | -3.11 |
|  |  | LOC111485078 | 2.19 | 1.13 |
|  |  | LOC111485079 | 0.16 | -2.63 |
|  |  | LOC111497144 | 0.05 | -4.32 |
|  | ***CYCD3*** | LOC111481144 | 3.42 | 1.77 |
|  |  | LOC111486045 | 0.33 | -1.60 |
|  |  | LOC111495987 | 0.21 | -2.27 |
| **Jasmonic acid** | ***JAR1*** | LOC111479305 | 3.76 | 1.91 |
|  | ***COI1*** | LOC111477426 | 2.24 | 1.17 |
|  |  | LOC111490898 | 2.53 | 1.34 |
|  | ***JAZ*** | LOC111492925 | 6.55 | 2.71 |
|  |  | LOC111467486 | 5.06 | 2.34 |
|  |  | LOC111494570 | 2.38 | 1.25 |
|  |  | LOC111485713 | 2.12 | 1.08 |
|  |  | LOC111488365 | 2.95 | 1.56 |
|  | ***MYC2*** | LOC111498362 | 3.10 | 1.63 |
|  |  | LOC111466936 | 0.34 | -1.56 |
| **Salicylic acid** | ***NPR1*** | LOC111467143 | 0.26 | -1.93 |
|  |  | LOC111495070 | 3.03 | 1.60 |
|  |  | LOC111482285 | 2.20 | 1.14 |
|  | ***TGA*** | LOC111470067 | 3.11 | 1.64 |
|  |  | LOC111466587 | 0.34 | -1.57 |
|  |  | LOC111469991 | 2.09 | 1.06 |
|  |  | LOC111471612 | 0.47 | -1.08 |
|  |  | LOC111489632 | 2.08 | 1.06 |
